# Immunization with a peptide mimicking lipoteichoic acid induces memory B cells in BALB/c mice

**DOI:** 10.1186/s12879-024-09262-8

**Published:** 2024-04-02

**Authors:** Xia-Yu Yi, Xiao-Rui Hou, Zhao-Xia Huang, Ping Zhu, Bei-Yi Liu

**Affiliations:** 1 Department of Immunology, School of Basic Medical Sciences, Southern Medical University, Guangzhou, Guangdong P.R. China; 2 Present Address: Department of Clinical Laboratory, The First People’s Hospital of Wuhu, Wuhu, Anhui P.R. China; 3 Present Address: Guangdong Provincial Engineering Research Center of Molecular Imaging, The Fifth Affiliated Hospital, Sun Yat-sen University, Zhuhai, Guangdong P.R. China

**Keywords:** *Staphylococcus aureus* (*S. aureus*), Lipoteichoic acid (LTA), Mimic peptide, Multiple antigenic peptide (MAP)

## Abstract

**Background:**

There is an urgent clinical need for developing novel immunoprophylaxis and immunotherapy strategies against *Staphylococcus aureus* (*S. aureus*). In our previous work, immunization with a tetra-branched multiple antigenic peptide, named MAP2-3 that mimics lipoteichoic acid, a cell wall component of *S. aureus*, successfully induced a humoral immune response and protected BALB/c mice against *S. aureus* systemic infection. In this study, we further investigated whether vaccination with MAP2-3 can elicit immunologic memory.

**Methods:**

BALB/c mice were immunized with MAP2-3 five times. After one month of the last vaccination, mice were challenged with heat-killed *S. aureus* via intraperitoneal injection. After a 7-day inoculation, the percentage of plasma cells, memory B cells, effector memory T cells, and follicular helper T cells were detected by flow cytometry. The levels of IL-6, IL-21, IL-2, and IFN-γ were measured by real-time PCR and ELISA. Flow cytometry results were compared by using one-way ANOVA or Mann-Whitney test, real-time PCR results were compared by using one-way ANOVA, and ELISA results were compared by using one-way ANOVA or student’s t-test.

**Results:**

The percentage of plasma cells and memory B cells in the spleen and bone marrow from the MAP2-3 immunized mice was significantly higher than that from the control mice. The percentage of effector memory T cells in spleens and lymphoid nodes as well as follicular helper T cells in spleens from the MAP2-3 immunized mice were also higher. Moreover, the levels of IL-6 and IL-21, two critical cytokines for the development of memory B cells, were significantly higher in the isolated splenocytes from immunized mice after lipoteichoic acid stimulation.

**Conclusions:**

Immunization with MAP2-3 can efficiently induce memory B cells and memory T cells.

**Supplementary Information:**

The online version contains supplementary material available at 10.1186/s12879-024-09262-8.

## Background

*Staphylococcus aureus* (*S. aureus*) is a common Gram-positive bacterium that can cause a wide spectrum of diseases, ranging from relatively mild skin infections to life-threatening infections, such as bacteremia and septic shock [1]. Since the emergence of antibiotic-resistant strains, such as methicillin-resistant *Staphylococcus aureus* (MRSA), *S. aureus* infection has become a growing threat to public health worldwide. As reported, 119,247 *S. aureus* bloodstream infections with 19,832 associated deaths occurred in the U.S. in 2017 [2]. Meanwhile, the cost and healthcare burden due to *S. aureus* infection also significantly increased [3]. An effective prophylactic vaccine needs to be developed.

Lipoteichoic acid (LTA) is an essential component of the cell wall that is expressed steadily on the surface of *S. aureus*. The basic structure of the LTA backbone is formed by conserved, unbranched, repeating units of 1,3-glycerolphosphate that are linked to the cytoplasmic membrane via a glycolipid anchor [4, 5]. Previous works have demonstrated that LTA is essential for the pathogenicity and survival of *S. aureus* under low-osmolarity conditions [4]. LTA also mediated host cell adhesion [6], biofilm formation [7] as well as penetration of the blood-brain barrier [8]. However, as a thymus-independent antigen (TI antigen), LTA is generally not considered a rational vaccine candidate.

In our previous work, MAP2-3, a tetra-branched multiple antigenic peptide (MAP) mimicking the epitope of LTA, was synthesized. MAP2-3 consists of four copies of the sequence HSGHKEDRQWCQHSGG, whose C-terminus is linked to a non-immunogenic and lysine-based dendritic scaffold [9]. Immunization with MAP2-3 induced *S. aureus* LTA-specific IgG antibodies, prolonged the survival, and decreased the bacterial burden in the organs of BALB/c mice, thereby protecting mice against *S. aureus* systemic infection. Moreover, passive immunization with polyclonal anti-MAP2-3 sera reduced bacterial load in the organs of mice with bacteremia and alleviated lung injury and skin lesions in mice models with *S. aureus* infection. After one month of the last immunization of this peptide, LTA-specific IgG antibody-secreting cells (ASCs) could be detected in splenocytes [9]. As a follow-up study of our previous work, we further investigated whether MAP2-3 immunization can induce the production of memory B cells.

## Methods

### LTA and MAPs

LTA from *S. aureus* (Cat No.2515) was purchased from Sigma. MAP2-3 (molecular weight: 7726.3) and the lysine-based, tetra-branched attaching backbone of MAP (named MAPctrl, molecular weight: 402.5) were synthesized with purity above 90% by Hybio Pharmaceutical (Shenzhen, China). MAPs were dissolved in endotoxin-free water (Sigma) at 50 mg/ml and diluted in sterile phosphate-buffered saline (PBS) when used in the assay.

### Antibodies

FITC-labeled anti-CD4 (clone: GK1.5), FITC-labeled anti-CD19 (clone: 6D5), PE-labeled anti-B220 (clone: RA3-6B2), PE-labeled anti-CD95 (clone: SA367H8), PE-labeled anti-CD44 (clone: IM7), PE/Cy7-labeled anti-IgD (clone:11-26c.2a), PE/Cy5-labeled anti-CD62L (clone: MEL-14), APC-labeled anti-IgG (clone: Poly4053), APC-labeled anti-CD138 (clone: Syndecan-1), APC-labeled anti-CD80 (clone: 16-10A1), and Pacific Blue-labeled anti-GL7 (clone: GL7) were purchased from BioLegend. CD16/CD32 purified antibody (clone: 93), and FITC-labeled anti-CD38 (clone: 90) were purchased from eBioscience.

### Preparation of bacteria

*S. aureus* (ATCC 25,923) was purchased from Wenzhou Kont Biology and Technology. The bacteria were grown in tryptic soy broth at 37 °C with 250 rpm shaking overnight. Cells were collected, washed, and diluted with sterile PBS to an appropriate concentration.

### Animal and ethics statement

BALB/c mice (5–6 weeks old, female) were purchased from the Experimental Animal Center, Southern Medical University, Guangzhou, China. All the animal experiments were approved by the Institutional Animal Care and Use Committee of Southern Medical University (permit number: L2015070) and carried out in strict accordance with national guidelines for animal welfare. The study was conducted in accordance with the ARRIVE guidelines and AVMA guidelines for the euthanasia of animals (2020) for reporting animal experiments.

### Immunization with MAPs and inoculation with *S. aureus*

**Fig. 1:**
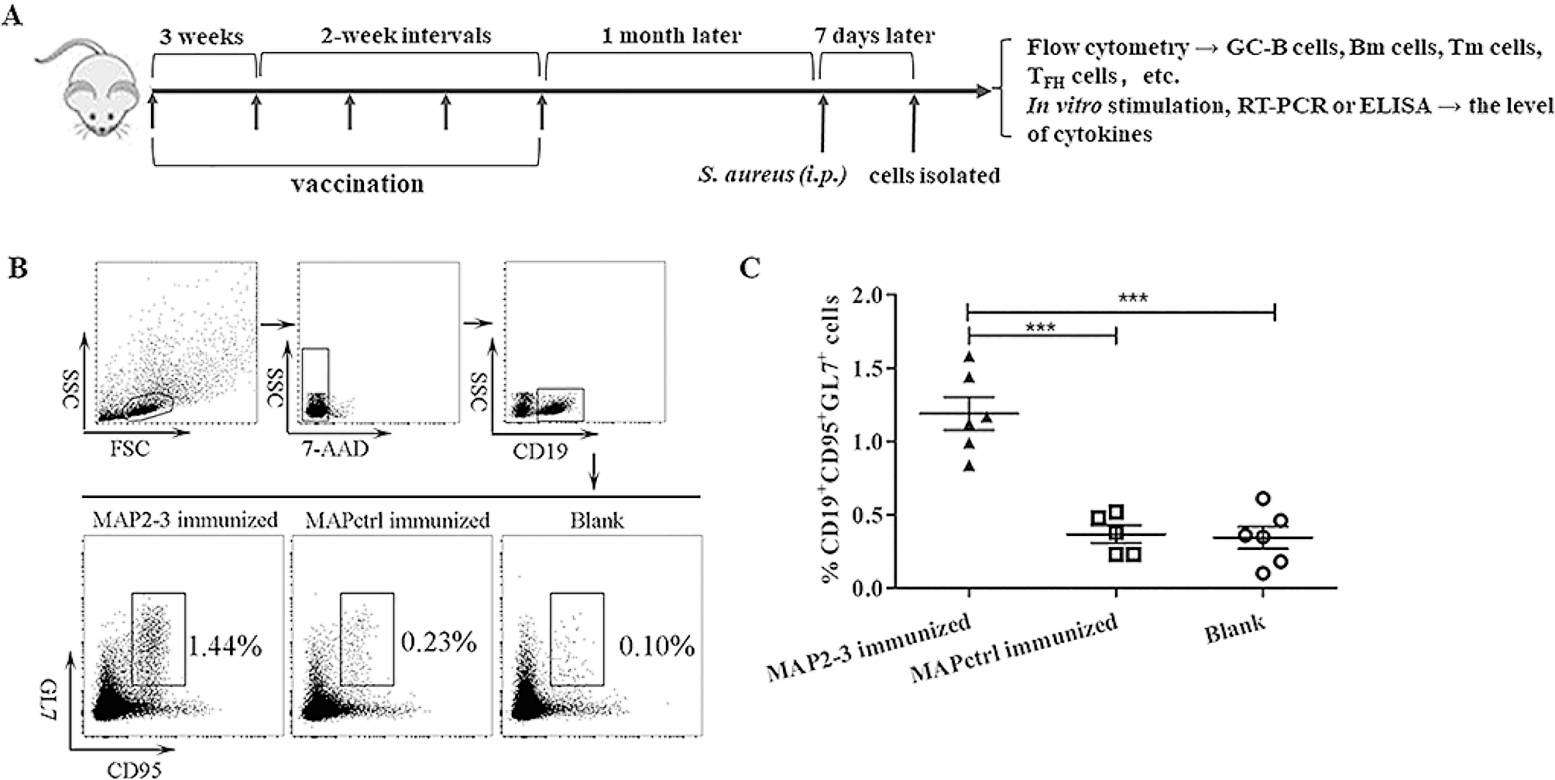
Diagram of the experimental design and identification of GC-B cells in the spleen. (**A**) Mice were immunized with MAP2-3 or MAPctrl 5 times. After one month of the last immunization, all mice were inoculated with heat-killed *S. aureus* (2 × 10^7^ CFU/mice) via *i.p.* The splenocytes, lymph node cells, and bone marrow cells were isolated 7 days after the inoculation. The percentage of different immune cells, including GC-B cells, plasma cells, memory B (Bm) cells, memory T (Tm) cells, etc. was identified by flow cytometry, respectively. Isolated splenocytes were stimulated with LTA or heat-killed *S. aureus* and the level of cytokines was determined by RT-PCR or ELISA. (**B**) Flow cytometry analysis of GC-B cells. Gating strategy and representative FACS of GC-B cells. (**C**) The percentage of GC-B cells in spleens. MAP2-3 *vs.* MAPctrl: *p* < 0.001; MAP2-3 *vs.* Blank: *p* < 0.001. *n* = 5–6 mice/group. *** *p* < 0.001

Vaccination was performed using the method based on our previous study [9]. Briefly, BALB/c mice were randomly divided into three groups: mice immunized with MAP2-3 (herein referred to as “MAP2-3 mice”), mice immunized with MAPctrl (MAPctrl mice), and mice without any peptide immunization used as a blank control. MAPs were injected into mice subcutaneously (100 µg /200 µl/ mouse) five times. The interval between the initial immunization and the second was 3 weeks, while the interval between the following booster immunization was 2 weeks. The first immunization was administered in Freund’s complete adjuvant (Cat No. F5881, Sigma) and the subsequent booster immunizations were administered in Freund’s incomplete adjuvant (Cat No. F5506, Sigma). One month after the last immunization, mice were injected intraperitoneally (*i.p*) with heat-killed *S. aureus* (2 × 10^7^ CFU/mouse) to mimic the bacterium infection. After seven days of challenge, mice were anesthetized with 1% pentobarbital sodium (50 mg/kg) by intraperitoneal injection, then euthanized by cervical dislocation. The splenocytes, bone marrow cells, and lymph node cells were isolated followed by flow cytometry analysis and *in vitro* assays (Fig. 1A).

### Isolation of splenocytes, bone marrow cells, and lymph node cells

The spleen and lymph nodes were aseptically excised and gently ground followed by filtering through a stainless mesh (size = 70 μm) in 10 ml RPMI 1640 media. Cell suspensions were pelleted and washed one time with 10 ml cold RPMI 1640 media. To lyse erythrocytes, 1×RBC lysis buffer (eBioscience, Cat No. 00-4300-54) was added for 5 min, followed by the addition of 20 ml of cold RPMI 1640 media to stop the lysis. After being washed two times with RPMI 1640, the erythrocyte-free cells were resuspended in the complete RPMI 1640 media.

Bone marrow cells were isolated using the protocol based on Kelly Roney’s work [10]. Briefly, the femur and tibia were isolated from the hind legs of mice. Any additional muscle or connective tissue attached to the bones was removed. An 18 G needle was then inserted into the bone and bone marrow cells were flushed into a tube. Clumps of bone marrow cells were gently dispersed and cell solutions were pipetted through a 70 μm cell strainer. Cells were then washed with PBS and lysed with 1 ml 1× RBC lysis buffer for 1 min. The lysis was stopped by adding 50 ml cold PBS and cells were pelleted after centrifugation at 1500 rpm at 4 °C for 10 min. Cells were washed with RPMI 1640 media and resuspended in an appropriate volume of cold RPMI 1640 media containing 10% calf serum.

### Flow cytometry

To reduce the non-specific binding of antibodies to Fc receptors expressed on the cell membrane, cells (2 × 10^6^ /ml) were first incubated with 1 µg/ml of anti-CD16/CD32 at room temperature for 10 min. Cells were then washed and incubated with specific antibodies for 30 min on ice in the dark. Samples were loaded on a BD LSR II flow cytometer and data were analyzed with FlowJo software (version X.0.7, Treestar, Inc). Dead cells were excluded by using 7-AAD in all flow cytometry experiments. Different immune cell types were identified by using specific cell surface markers. GC-B cells and plasma cells were defined as CD19^+^ CD95^+^ GL7^+^ cells and B220^−^ CD138^+^ cells, respectively. Memory B cells were identified using two gating methods: compared with naïve B cells, memory B cells express membrane IgG (mIgG) or membrane IgG1 (mIgG1) but not membrane IgM (mIgM) [11, 12], thus, CD19^+^ IgM^−^ IgG^+^ or CD19^+^ IgM^−^ IgG1^+^ were used as surface markers for memory B cells. Besides, memory B cells but not the plasma cells or GC-B cells express B220 [12] and CD38 [13], therefore our second method for identifying memory B cells was to use B220^+^ CD38^+^ IgG^+^ IgD^−^ GL7^−^ as their surface markers. Other immune cell types including follicular helper T cells (T_FH_, CD4^+^ CXCR5^+^ PD-1^+^) [14], effector memory T cells (T_EM_, CD4^+^ CD44^+^ CD62L^−^), and central memory T cells (T_CM_, CD4^+^ CD44^+^ CD62L^+^) were also identified.

### RNA isolation and real-time PCR

Splenocytes (4 × 10^5^/well) were added to the 96-well cell culture plates. The cells were stimulated with LTA derived from *S. aureus* (10 µg/ml) at 37 °C for 24 h. Total RNA was extracted from splenocytes and purified using RNAiso Plus (TaKaRa, Cat No. 9109). cDNA was converted from 1 µg total RNA using the RevertAid First Strand cDNA synthesis kit (Fermentas, Cat No. K1622). Real-time PCR analysis was performed using the Rotor-Gene1000 system (Corbett Research, Bath, UK). Briefly, cDNA was serial diluted and PCR-amplified with SYBR Green PCR mix (Toyobo, Cat No. QPK-201) in triplicates. The program for amplification was set as 1 cycle of 95 °C for 30 s followed by 40 cycles of 94 °C for 30 s, 55 °C for 30 s, and 72 °C for 60 s. The glyceraldehyde 3-phosphite dehydrogenase (GAPDH) gene was used as an endogenous control to normalize the differences in the amount of total RNA present in the samples. Primer sequences are as follows: IL-21 sense, 5’-ACCCCTGGCTTTCACTGTTT-3’; anti-sense, 5’-CTGAGGCTGGAGCTAGCAGA-3’; IL-6 sense, 5’-TCCAGAAACCGCTATGAAGTT-3’; anti-sense, 5’-TTCATACAATCAGAATTGCCATT-3’ and GAPDH sense, 5’-TGTGTCCGTCGTGGATCTGA-3’; anti-sense, 5’-TTGCTGTTGAAGTCGCAGGA-3’. Data were analyzed using the 2^−△△CT^ method and mRNA levels were calculated as the fold change compared to the control group.

### Detection of cytokine in splenocytes incubated with heat-killed *S. aureus*

Splenocytes (4 × 10^5^/well) were incubated with heat-inactivated *S. aureus* (2 × 10^4^ CFU/well) at 37 °C for 24 h. The supernatant was then collected and the level of IL-6, IL-2, and IFN-γ was determined by ELISA (BioLegend) according to the manufacturer’s instructions. All measurements were analyzed in triplicates.

### Statistical methods

Numeric measurements were expressed as means ± standard error of the mean (SEM). Each experiment under the same condition was repeated two or three times. For analyzing flow cytometry data, if the data conformed to normal distribution and homogeneity of variance, the comparison was performed using one-way ANOVA with the least significant difference (LSD) method. If the data conformed to normal distribution but not homogeneity of variance, the comparison was performed using one-way ANOVA with Dunnett’s T3 test. If the data conformed to neither normal distribution nor homogeneity of variance, the comparison was performed using a Mann-Whitney test. The concentration of cytokines (IL-6, IL-2, and IFN-γ) was compared between groups using either one-way ANOVA or Student’s t-test. Expression of cytokines (IL-6 and IL-21) was compared between groups using one-way ANOVA with Dunnett’s T3 test. The significance of the test will be assessed at alpha = 0.05.

## Results

### Immunization with MAP2-3 promoted the production of GC-B cells and plasma cells after heat-killed *S. aureus* inoculation

In response to an antigen challenge, germinal centers (GCs) first form in the secondary lymphoid organ, including the spleen and lymph nodes. Antigen-specific B cells subsequently migrate into the GCs and form GC-B cells [15, 16]. With the stimulation of specific antigens and help from T_FH_, some GC-B cells can differentiate into plasma cells or memory B cells [17]. Since we previously found that MAP2-3 immunization induced LTA-specific IgG antibodies and LTA-specific antibody-secreting cells (ASCs) [9], we first investigated whether MAP2-3 immunization can induce the production of GC-B cells after heat-killed *S. aureus* challenge. The percentage of GC-B cells (CD19^+^ CD95^+^ GL7^+^) in MAP2-3 mice was significantly higher than that in control mice (Fig. 1B and C), indicating that MAP2-3 immunization induces the precursors of plasma cells.

**Fig. 2:**
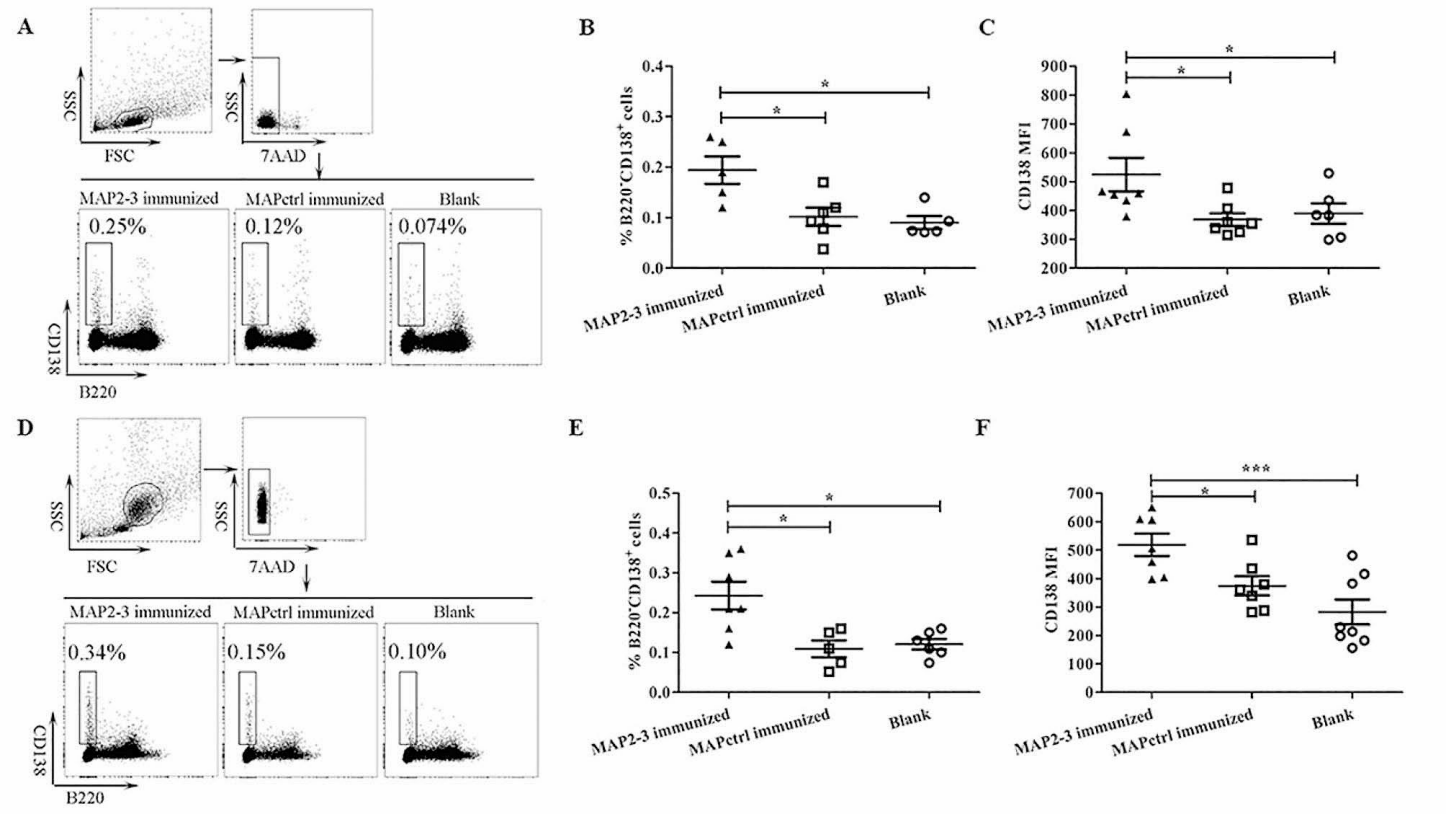
Detection of plasma cells (B220^−^ CD138^+^ cells) in the spleen and bone marrow. (**A**) Gating strategy and representative FACS of plasma cells in the spleen. (**B**) The percentage of plasma cells in the spleen. MAP2-3 *vs.* MAPctrl: *p* = 0.022; MAP2-3 *vs.* Blank: *p* = 0.016. (**C**) MFI of CD138 was examined using B220^−^ CD138^+^ cells in the spleen. MAP2-3 *vs.* MAPctrl: *p* = 0.015; MAP2-3 *vs.* Blank: *p* = 0.038. *n* = 5–7 mice/group. (**D**) Gating strategy and representative FACS of plasma cells in the bone marrow. (**E**) The percentage of plasma cells in the bone marrow. MAP2-3 *vs.* MAPctrl: *p* = 0.026;MAP2-3 *vs.* Blank: *p* = 0.034. (**F**) MFI of CD138 was examined using B220^−^ CD138^+^ cells in the bone marrow. MAP2-3 *vs.* MAPctrl: *p* = 0.021; MAP2-3 *vs.* Blank: *p* = 0.0004. *n* = 5–8 mice/group. *p*-values: * *p* < 0.05, *** *p* < 0.001

We next examined the percentage of plasma cells (B220^-^ CD138^+^) in splenocytes after a 7-day *S. aureus* inoculation. Compared with MAPctrl mice or blank control mice, the percentage of plasma cells in the MAP2-3 mice was significantly higher (Fig. 2A and B). Consistently, the mean fluorescence intensity (MFI) of CD138^+^ plasma cells in the spleen of MAP2-3 mice was also significantly higher (Fig. 2C). As reported, some plasma cells differentiated from GC-B cells can exit the germinal center and migrate to the bone marrow to secret high-affinity antibodies [15]. Based on this knowledge, we further examined whether the percentage of plasma cells in bone marrow increased after challenge. As shown in Fig. 2D-F, the percentage of B220^-^ CD138^+^ plasma cells, as well as the MFI of CD138^+^ plasma cells isolated from bone marrow, was significantly higher in MAP2-3 mice than that from the control mice. All these results indicate that MAP2-3 immunization induced the production of plasma cells in the spleen and bone marrow when the mice were challenged with heat-activated *S. aureus*.

### MAP2-3 immunization induced the production of memory B cells in the spleen and bone marrow

**Fig. 3:**
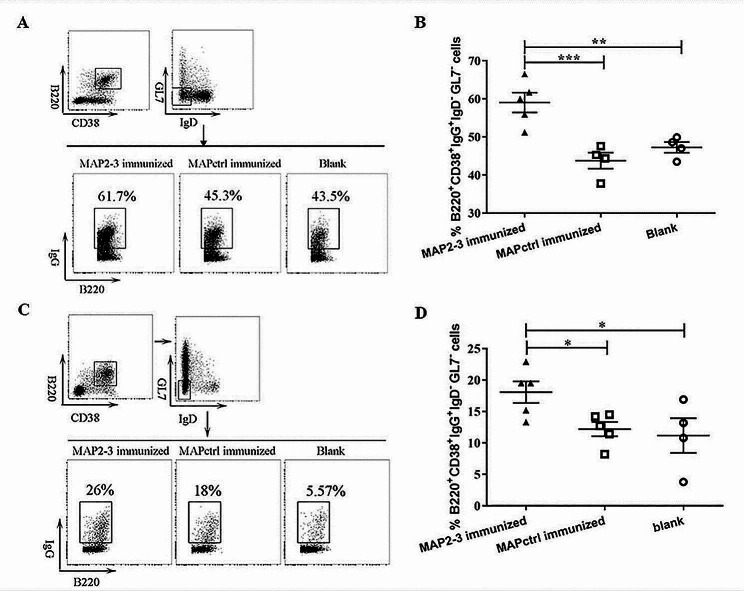
Detection of memory B cells in the spleen and bone marrow from the immunized mice. (**A**) Gating strategy and representative FACS of memory B cells in the spleen. (**B**) The percentage of memory B cells in the spleen. MAP2-3 *vs.* MAPctrl: *p* = 0.00057; MAP2-3 *vs.* Blank: *p* = 0.003. *n* = 4–5 mice/group. (**C**) Gating strategy and representative FACS of memory B cells in the bone marrow. (**D**) The percentage of memory B cells in the bone marrow. MAP2-3 *vs.* MAPctrl: *p* = 0.04; MAP2-3 *vs.* Blank: *p* = 0.026. *n* = 4–5 mice/group. Data are expressed with mean ± SEM. *p*-values: * *p* < 0.05, ** *p* < 0.01, *** *p* < 0.001

In addition to inducing specific antibodies, immunization with a vaccine candidate can stimulate the production of memory B cells when re-exposure to the specific antigen. As shown in Fig. 3, the percentage of memory B cells (B220^+^ CD38^+^ IgG^+^ IgD^−^ GL7^−^) was significantly higher in both spleen (Fig. 3A and B) and bone marrow (Fig. 3C and D) after one month of the last immunization followed by *S. aureus* inoculation. Moreover, the percentage of IgG^+^ or IgG1^+^ memory B cells (IgG^+^/IgG1^+^ CD19^+^ B220^+^ IgM^−^) in the spleen of MAP2-3 mice was also significantly higher 5 days after the last immunization (supplementary Fig. S1.). Taken together, these results indicated that memory B cells can be induced by MAP2-3 immunization.

### MAP2-3 immunization induced CD80 expression on the surface of memory B cells and induced T_FH_ cells in the spleen

**Fig. 4:**
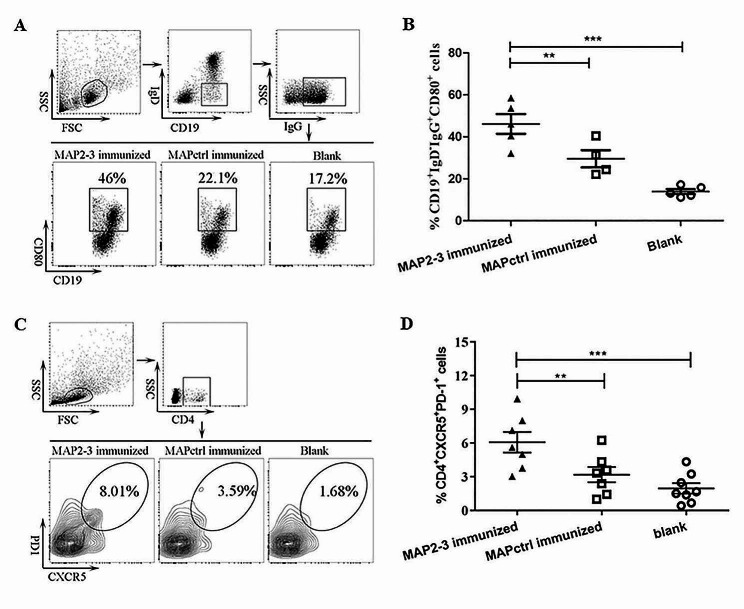
Detection of CD80^+^ memory B cells and T_FH_ cells from the immunized mice. (**A**) Gating strategy and representative FACS of memory B cells with CD80 expressed on the surface. (**B**) The percentage of memory B cells with CD80 expressed on the surface. MAP2-3 *vs.* MAPctrl: *p* = 0.009; MAP2-3 *vs.* Blank: *p* < 0.001. *n* = 4–5 mice/group. (**C**) Gating strategy and representative FACS of T_FH_ cells in the spleen. (**D**) The percentage of T_FH_ cells in the spleen. MAP2-3 *vs.* MAPctrl: *p* = 0.009895; MAP2-3 *vs.* Blank: *p* = 0.000467. *n* = 7–8 mice/group. *p*-values: ** *p* < 0.01, *** *p* < 0.001

CD80 is one of the critical markers expressed on memory B cells in mice [12], which regulates B-T interactions [18] and promotes follicular T cell generation [19]. Thus, we examined the expression of CD80 on the surface of IgG^+^ memory B cells and found that the percentage of CD19^+^ IgD^−^ IgG^+^ CD80^+^ memory B cells in splenocytes from MAP2-3 mice was significantly higher than that from the control mice (Fig. 4A and B).

Different from effector helper T cells, such as Th1, Th2, or Th17, which predominantly migrate from the lymphoid tissue to sites of inflammation or infection, T_FH_ cells mainly reside in the spleen and lymph nodes and play an important role in promoting GC-B cells to differentiate into memory B cells and long-lived plasma cells [20]. As shown in Fig. 4C and D, the percentage of CD4^+^ CXCR5^+^ PD-1^+^ T_FH_ cells in spleens from MAP2-3 mice was significantly higher than that from the control mice after the *S. aureus* challenge.

### MAP2-3 immunization increased the percentage of effector memory T cells (T_EM_ ) in the spleen or lymph nodes after *S. aureus* inoculation

**Fig. 5:**
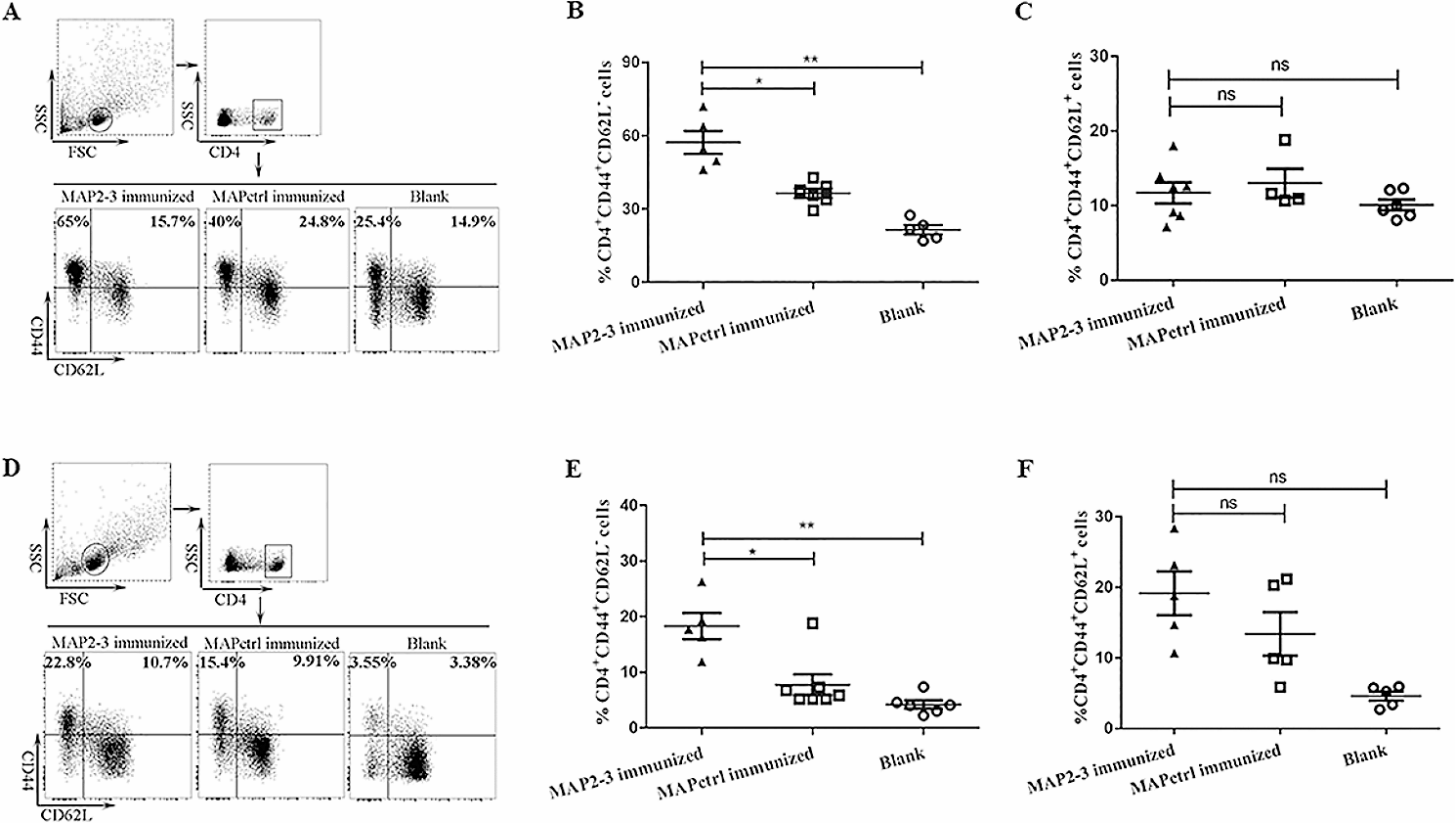
Detectionof memory T cells in the spleen and lymphoid node from the immunized mice. (**A**) Gating strategy and representative FACS of memory T cells in the spleen. (**B**) The percentage of T_EM_ cells in the spleen. MAP2-3*vs.* MAPctrl: *p* = 0.022; MAP2-3*vs.* Blank: *p* = 0.002. (**C**) The percentageof T_CM_ cells in the spleen. There is no difference between groups (*p*> 0.05). n = 4-7 mice/group. (**D**) Gating strategy and representative FACSof memory T cells in the lymphoid node. (**E**) The percentage of T_EM_cells in the lymphoid node. MAP2-3 *vs.* MAPctrl:*p* = 0.019; MAP2-3 *vs.*Blank: *p* = 0.006. (**F**) The percentageof T_CM_ cells in the lymphoid node. There is no difference betweengroups (*p *> 0.05). n = 5-7mice/group. *p*-values: * *p* < 0.05, ** p < 0.01, ns *p > *0.05.

The frequency of memory T cells in peripheral immune organs, including the spleen and lymph nodes, was further investigated. The percentage of T_EM_ (CD4^+^ CD44^+^ CD62L^−^) from MAP2-3 mice was significantly higher than the controls in both spleens (Fig. 5A and B) and lymphoid nodes (Fig. 5D and E). However, there is no significant difference in the percentage of T_CM_ (CD4^+^ CD44^+^ CD62L^+^) among the three groups in both the spleen (Fig. 5A and C) and lymphoid nodes (Fig. 5D and F).

### Stimulation with heat-killed *S. aureus* or LTA increased the level of interleukin-6 (IL-6) in the splenocytes of MAP2-3 mice

**Fig. 6:**
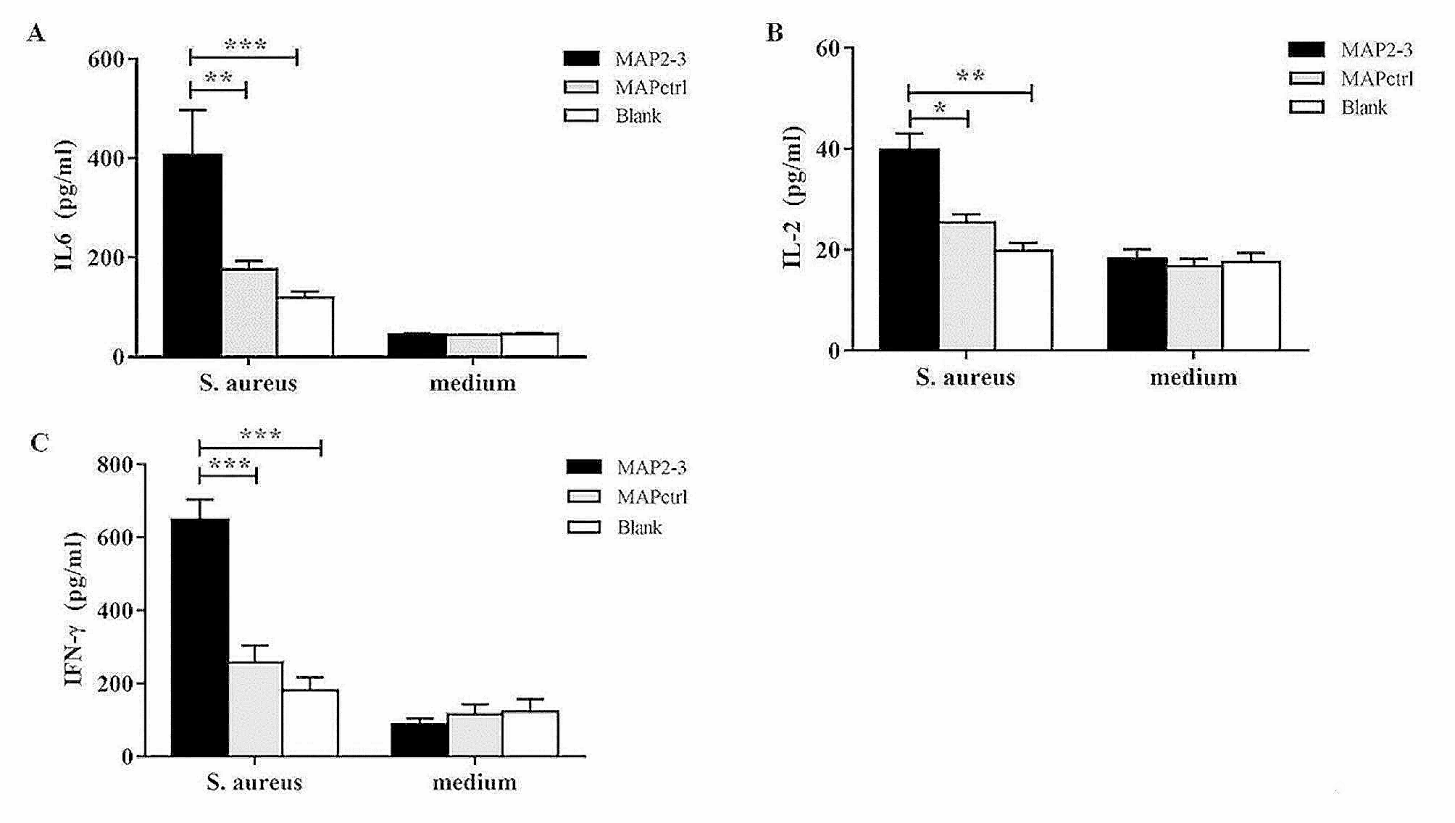
Detectionof cytokines in splenocytes stimulated by heat-killed *S. aureus*. Afterone month of the last immunization, all mice were inoculated with heat-killed *S.aureus* for 7 days. Isolated splenocytes were stimulated with heat-killed *S.aureus* (2×10^4^ CFU/well) for 24 h. The concentrationof IL-6 (A), IL-2 (B), and IFN-γ (C) wasmeasured by ELISA. (**A**) IL-6, MAP2-3 *vs.* MAPctrl: *p *= 0.0065; MAP2-3 *vs.* Blank: p < 0.001. (**B**) IL-2, MAP2-3 *vs.* MAPctrl: *p* = 0.017; MAP2-3*vs.* Blank: *p *= 0.003. (**C**) IFN-γ, MAP2-3 *vs.* MAPctrl: *p* <0.001;MAP2-3 *vs.* Blank: *p* < 0.001. n = 6-10 mice/group. *p*-values:* p < 0.05, ** *p* < 0.01, *** *p* < 0.001.

Since cytokines are crucial to plasma cells and memory B cells development, the results showed that the concentrations of IL-6, IL-2, and IFN-γ secreted from splenocytes in the MAP2-3 mice were significantly higher than that from the control mice after *in vitro* heat-killed *S. aureus* stimulation (Fig. 6A-C). In addition, IL-6 and IL-21 are two important cytokines for T_FH_ cells, plasma cell differentiation, and memory B cells production [20–22], we next measured the levels of IL-6 and IL-21 in the isolated splenocytes stimulated with LTA *in vitro*. The results showed that the expression of these two cytokines was significantly higher in the MAP2-3 mice as compared to that in the control mice (supplementary Fig. S2).

## Discussion

In this study, we provide evidence using *in vitro* experiments that immunization with MAP2-3, a peptide mimicking LTA, could induce immunologic memory after one month of the last immunization followed by heat-killed *S. aureus* inoculation. First of all, the percentage of plasma cells in the bone marrow and spleen significantly increased. Second, the proportion of memory B cells in the spleen and the bone marrow from the MAP2-3 mice was significantly increased. Third, the percentage of effector memory T cells in the lymph node from the MAP2-3 mice was also significantly increased.

Previous studies have shown that T_FH_ cells play an important role in the differentiation of GC-B cells and the formation of memory B cells [23, 24]. IL-21 secreted from T_FH_ cells is one of the key cytokines for the generation of long-lived plasma cells [25, 26]. In addition, IL-6 plays a role in the formation of GC, promotes antibody production, and maintains T_FH_ cells [27]. Eto D. and colleagues found that the coordination between IL-6 and IL-21 was necessary for T_FH_ cell formation [22]. IFN-γ also effectively activates mononuclear macrophages and promotes the production of IgG antibodies, thereby playing an important anti-infective function in bacteremia and sepsis caused by *S. aureus* infection [28]. Consistent with the literature, our work shows that the proportion of T_FH_ cells in the spleen of MAP2-3 mice was significantly higher than the controls after the *S. aureus* challenge. Cytokines, including IL-21, IL-6, IL-2, and IFN-γ were significantly induced in the MAP2-3 mice after *in vitro* LTA or *S. aureus* stimulation (Fig. 6 and supplementary Fig. S2). Other cytokines such as IL-4 and TGF-β are also important for B cell proliferation, differentiation, and class switching [14]. However, we failed to detect the induction of these cytokines after LTA or *S. aureus* stimulation (data not shown). We speculate that the induction of different cytokines is time-dependent and antigen-dose-dependent. Further experiments will be performed to assess the expression of more cytokines (especially secreted by antigen-specific T cells) and their roles in immune responses in mice with MAP2-3 immunization.

We have previously shown that MAP2-3 immunization protected mice from the infection of live *S. aureus* [9]. In this study, heat-killed *S. aureus* was used as a surrogate since inactivated bacteria still retain their components and immunogenicity. Previous studies also used inactivated bacteria to determine the immunological response, especially in the flow cytometry assays [29, 30]. Cruciani M’s work further demonstrated that both live *S. aureus* and inactivated bacteria induce the expression of CD86 and MHC II molecules on dendritic cells and there are no differences in terms of dendritic cell viability and maturation [31]. In future work, we will compare the immune response of MAP2-3 immunized mice to heat-killed *S. aureus* versus live ones.

Although we demonstrated that memory B cells and related cytokines were induced after peptide immunization, evidence is lacking to prove that these cells are specific for *S. aureus*. In future work, we will examine the induction of antigen-specific plasma cells, memory B cells, and T cells after stimulation using fluorescein-labeled MAP2-3 peptide, LTA or *S. aureus*. We will also examine the specificity of peptide-induced immune response by comparing different *S. aureus* strains and other bacteria.

Peptide-based vaccines are produced almost exclusively using chemical synthetic approaches. This production procedure is simple and fast and there is no biological contamination that may induce allergic responses compared with inactivated vaccine and recombinant protein-based vaccine [32]. However, peptides are poor immunogens on their own and are susceptible to enzymatic degradation compared with protein-derived antigens. Peptide used as a vaccine needs the assistance of an adjuvant or delivery system. Alum, the only widely used adjuvant for humans, is not suitable for peptide antigens due to its weak adjuvant potency [33]. Our previous work also showed that immunization with MAP2-3 emulsified with alum could not effectively induce a humoral immune response (data not shown). In contrast, immunization of MAP2-3 emulsified with Freund’s adjuvant could induce LTA-specific IgG antibodies [9], memory B cells, and memory T cells. However, this adjuvant is not suitable for human vaccination due to its toxicity. Some new adjuvants such as squalene-based emulsions including MF59 and AS03 have been licensed for human application [34, 35]. In addition, alum plus TLR or CpG-ODN and liposomal plus CpG-ODN formulation can elicit a strong humoral immune response [36]. New antigen delivery systems such as polymers [37] and nanoparticles [38] have been recently used for promoting the uptake, transport, and presentation of antigens to APCs. In future experiments, we will examine the effect of novel adjuvants and delivery systems on the immunogenicity of MAP2-3.

## Conclusions

Memory B cells in the spleen and bone marrow as well as effector memory T cells in the spleen and lymphoid nodes can be induced after one month of immunization with an LTA-mimicking peptide, MAP2-3.

### Electronic supplementary material

Below is the link to the electronic supplementary material.

Supplementary Material 1

Supplementary Material 2

## Data Availability

All data generated or analyzed during this study are included in this article and its supplementary information files.

## Abbreviations

7-AAD: 7-aminoactinomycin D
ASCs: antibody-secreting cells
ATCC: American type culture collection
APC: Allophycocyanin
APCs: antigen presenting cells
Bm: memory B cells
CD: cluster of differentiation
cDNA: complementary DNA
CFU: colony-forming units
CXCR5: C-X-C chemokine receptor type 5
ELISA: enzyme linked immunosorbent assay
FACS: fluorescence activated cell sorting
Fc: fragment crystallizable
FCM: flow cytometry
FITC: fluorescein isothiocyanate
GAPDH: Glyceraldehyde 3-phosphite dehydrogenase
GC: germinal center
GC-B cells: germinal center B cells
h: hour
IL-6: interleukin 6
IL-21: interleukin 21
IL-2: interleukin 2
IFN-γ: interferon gamma
*i.p*: intraperitoneally
mIgD: membrane immunoglobulin D
mIgG/mIgG1: membrane immunoglobulin G or G1
mIgM: membrane immunoglobulin M
LTA: lipoteichoic acid
LSD: the least significant difference
MAP: multiple antigenic peptide
MFI: the mean fluorescence intensity
mg: milligram
min: minute
ml: milliliter
mRNA: messenger RNA
MRSA: methicillin-resistance *Staphylococcus aureus*
OD: optical density
PBS: phosphate-buffered saline
PD-1: programmed cell death-1
PE: Phycoerythrin
PE/Cy5: Phycoerythrin/Cyanine 5
PE/Cy7: Phycoerythrin/Cyanine 7
RBC: red blood cell
rpm: revolutions per minute
s: second
RT-PCR: real-time polymerase chain reaction
*S aureus*: *Staphylococcus aureus*
SEM: standard error of the mean
TI-antigen: thymus-independent antigen
T_CM_: central memory T cells
T_EM_: effector memory T cells
T_FH_: follicular helper T cells
Th: helper T cells
µl: microliter
µg: microgram
µm: micrometer
