# Supplementary material for: Immunization with a peptide mimicking lipoteichoic acid induces memory B cells in BALB/c mice
Source: BMC Infect Dis. 2024 Apr 2;24:371. doi: 10.1186/s12879-024-09262-8 (PMC10986077; doi:10.1186/s12879-024-09262-8)
Supplement: Supplementary file 1 — Supplementary Material 1 [file 12879_2024_9262_MOESM1_ESM.docx]

**Supporting Information 1**

**

**

**Supplementary Fig. S1. Detection of IgG^+^ or IgG1^+^ memory B cells after 5 days of immunization.**

After 5 days of the last immunization, the percentage of IgG^+^ memory B cells (A and B) and IgG1^+^ memory B cells (C and D) were measured by flow cytometry. (A) Gating strategy and representative FACS of the CD19^+^ B220^+^ IgM^-^ IgG^+^ memory B cells in the spleens. (B) The percentage of IgG^+^ memory B cells. MAP2-3 *vs.* MAPctrl: *p* = 0.028；MAP2-3 *vs.* blank: *p* = 0.012. n=3-4 mice/group. (C) Gating strategy and representative FACS of CD19^+^ B220^+^ IgM^-^ IgG1^+^ memory B cells in the spleen. (D) The percentage of IgG1^+^ memory B cells. MAP2-3 *vs.* MAPctrl: *p* = 0.013；MAP2-3 *vs.* blank: *p* = 0.002. n= 5-6 mice/group. *p*-values: * *p* < 0.05, ** *p* < 0.01.
