# Supplementary material for: Immunization with a peptide mimicking lipoteichoic acid induces memory B cells in BALB/c mice
Source: BMC Infect Dis. 2024 Apr 2;24:371. doi: 10.1186/s12879-024-09262-8 (PMC10986077; doi:10.1186/s12879-024-09262-8)
Supplement: Supplementary file 2 — Supplementary Material 2 [file 12879_2024_9262_MOESM2_ESM.docx]

**Supporting information 2.**


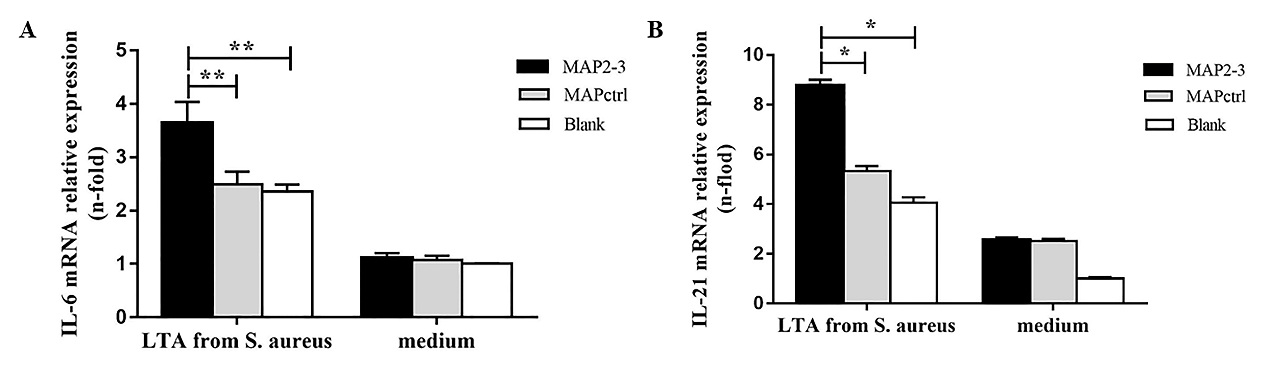


**Supplementary Fig. S2. Detection of the cytokines in splenocytes stimulated by LTA.**

After one month of the last immunization, all mice were inoculated with heat-killed *S. aureus* for 7 days. Isolated splenocytes were stimulated with LTA (10 μg/ml) for 24 h. The level of IL-6 (A) and IL-21 (B) was measured by real-time PCR. (A) IL-6, MAP2-3 *vs.* MAPctrl: *p* = 0.008; MAP2-3 *vs.* Blank: *p* = 0.009. (B) IL-21, MAP2-3 *vs.* MAPctrl: *p* = 0.012; MAP2-3 *vs.* Blank: *p* = 0.017. n = 3-5 mice/group. * *p* < 0.05, ** *p* < 0.01.
